# Experimental Investigation on the Low Velocity Impact Response of Fibre Foam Metal Laminates

**DOI:** 10.3390/ma14195510

**Published:** 2021-09-23

**Authors:** Patryk Jakubczak, Magda Droździel, Piotr Podolak, Jesus Pernas-Sánchez

**Affiliations:** 1 Department of Materials Engineering, Faculty of Mechanical Engineering, Lublin University of Technology, Nadbystrzycka 36, 20-618 Lublin, Poland; m.drozdziel@pollub.pl (M.D.); p.podolak@pollub.pl (P.P.); 2 Department of Continuum Mechanics and Structural Analysis, University Carlos III of Madrid, Avda. de la Universidad 30, 28-911 Madrid, Spain; jpernas@ing.uc3m.es

**Keywords:** fibre metal laminates, foam laminates, composite failure, impact

## Abstract

The combination of fibre metal laminates (FML) and sandwich structures can significantly increase the performance under impact of FMLs. The goal of this work was to create a material that will combine the superior properties of FMLs and foam sandwich structures in terms of the impact resistance and simultaneously have lower density and fewer disadvantages related to the manufacturing. An extensive impact testing campaign has been done using conventional fibre metal laminates (carbon- and glass-based) and in the proposed fibre foam metal laminates to assess and compare their behaviour. The main difference was observed in the energy absorption mechanisms. The dominant failure mechanism for fibre foam laminates is the formation of delaminations and matrix cracks while in the conventional fibre metal laminate the main failure mode is fibre cracking due to high local stress concentrations. The reduction in the fibre cracking leads to a better after-impact resistance of this type of structure improving the safety of the structures manufactured with these materials.

## 1. Introduction

Fibre metal laminates (FMLs) consist of a hybrid structure obtained by alternating metal layers and fibre reinforced laminate [1]. They are widely used in the aerospace and automotive industry due to their low density and simultaneous high fatigue strength [2], impact resistance [3,4], low crack growth rates, as well as corrosion resistance in comparison to classic metal alloys [5,6,7]. Due to the use of fibre metal laminates in fuselages, among other applications, one of the most important issues related to the reliability of these structures is their impact resistance [8,9,10]. The low–velocity impact in fibre metal laminates causes failure modes such as buckling, initiation and propagation of delamination and also matrix and fibre cracks [9,10,11]. Despite the good durability of metal in instances of perforation, there is a problem of fibre cracking even at relatively low impact energies (up to 10 J) [3,12]. This is particularly crucial when using carbon fibres in the fibre metal laminates in which the strain to failure is smaller than that of glass fibres [9]. Literature data shows that carbon-based laminates are characterized by lower impact energy to the first damage than laminates based on glass fibres (GLARE), which has been confirmed in numerous studies [4,6,13]. In [11] the authors observed that delaminations and even fibre cracks occurred at energies as low as 5 and 7.5 J, respectively. The early damage is interplay matrix cracking and it is caused by transverse impacts, and their main consequence is the formation and propagation of delamination [4]. Due to the fact that FML laminates are characterized by high buckling resistance, it is more advantageous to enforce the absorption of impact energy by the delamination mechanism than by the fibre cracking, which is important in terms of post-impact behaviour. e.g., at compression after impact (CAI) [14,15].

In addition to FMLs, other types of structures are being developed to improve the impact resistance in different aviation applications. One of the alternatives for FML materials used in aerospace are sandwich composite structures. They are characterized by high resistance to impact and the ability to absorb energy [16,17]. Foam core structures have high crushability [18], are lightweight [19], and have a good energy absorption capacity [20]. Many researchers have investigated the impact resistance of sandwich composites with foam cores [21,22,23,24]. Schubel et al. [25] investigated sandwich panels consisting of woven—carbon/epoxy face sheets and a PVC foam core, which were subjected to impact. The authors reported that foam core structures, despite a higher impact energy, did not show an increase in the damaged area in comparison to the results seen in quasi–static tests, however, this phenomenon was not explained. Anderson and Madenci [26] showed the behaviour of sandwich composites subjected to low-velocity impacts. They studied sandwiches with graphite/epoxy face sheets comparing foam and honeycomb cores. Their results showed that a higher foam core density, but also thicker face layers, provide an enhancement in energy absorption and impact resistance. Long et al. [27] tested sandwich plates with carbon/epoxy composite skins and polyurethane foam cores under different impact energies. They observed different intra- and interlaminar damage modes such as matrix cracks, delaminations and foams fractures in the core. Moreover, they noticed that structures with a hard core are more prone to delaminations than those with a soft core. However, this distinction became irrelevant when penetration and perforation of the skins occurred. The main energy absorption of the core is through crushing and delaminations, which mainly happen before perforation. Hazizan and Cantwell [28] explained that if the core is brittle the initial failure is shear cracking through the foam thickness, and if the foam is tougher, the initial damage observed is a buckling failure in the top skin, whereas, Faidzi et al. [29] showed that using PVC foams in composite materials improved the ability to absorb energy because of progressive foam crushing and cracking. Similar conclusions can be found in the other studies such as Ren et al. [19] and Elamin et al. [30]. Caprino and Teti [31] carried out impact tests on panels made of a foam core and glass fibre skins. They summed up that the core density and the core thickness influence the impact resistance of sandwich structures, and the maximum force rises with increasing foam density. Similar conclusions were described in [32], in which the authors concluded that the foam core density significantly affects the structural response to a low velocity impact. The damage area in the tested samples decreases with increasing core density and simultaneously influences the enhancement in maximum force and energy absorption. Al–Shamary et al. [20] experimentally investigated the impact resistance of sandwich structures made of foam cores with different configurations and glass face skins. The authors revealed that an increase in core thickness did not prevent the formation of delaminations, but did reduce slightly the damage-affected area. Abbasi and Nia [33] experimentally and numerically investigated laminates consisting of aluminium layers and foam cores under high–velocity impact conditions. Their results showed that the core layering increases the ballistic limit velocity of the sandwich structures. Moreover, removal of the foam core led to a 32% reduction in the ballistic limit velocity.

Despite the fact that foam sandwich laminates are characterized by a relatively high energy absorption due to the presence of a foam component, they are characterized by low resistance to fatigue as well as low energy absorption capacity, or negligible strength in the third direction [34,35,36,37]. Moreover, as it was shown above, sandwich structures are vulnerable to low-velocity impacts. Foam sandwich laminates have different failure mechanisms depending on the impact energy value. The main failure modes include matrix cracking, delaminations, foam crushing, or face–core debonding and perforation [27,32,38]. Failure in sandwich panels is sensitive to the main constituents of the skins and the core, the adhesives used, the skin ply sequences and other factors [29].

Based on the state of art related to the impact features of fibre metal laminates and sandwich structures, it can be assessed that there is still a significant potential for improving their impact resistance. It was observed that FML laminates have unique properties related to the combination of metal and composites, but also unique features provided by the combination of foam cores with the composite. It seems that the combination of both components with special properties into one hybrid structure is justified from the point of view of the mechanics and physics of the phenomena occurring during an impact. The purpose of the present work is to design a hybrid FML structure, containing, besides composite and metal layers a polyester foam layer infiltrated with resin (fibre foam metal laminate of FFL). The goal was to create a material that will combine the superior properties of FMLs and foam sandwich structures in terms of the impact resistance and simultaneously will have lower density and fewer disadvantages related to their manufacture. In the case of the manufacturing of FMLs it is impossible to remove excess resin from the prepreg due to the external aluminum layers. This makes a difficult to removing the porosity and establish a proper resin content. The elastic foam inside the laminate provides the possibility to use excess resin, forming a coherent infiltrated layer with a thickness similar to that of the metal sheet.

It was assumed that the devised FFL hybrid structure is not simply a structure with another directly added foam layer. In a conventional FML structure, the inner metal layer was replaced with a foam structure, ensuring its complete coherence with the adjacent composite layers by resin infiltration of the foam. Aside from the impact resistant improvement of the FML due to the addition of a foam core (especially fibre cracking in the low energy range) the reduction of density, corrosion resistance and low moisture absorption capacity remain unchanged as in classic fibre metal laminates. Laminates based on aluminum, carbon and glass fibres and polyester foam were used to assess the low velocity impact resistance. Quantitative and qualitative criteria, including force changes with respect to material thickness and density, were used to evaluate and compare the impact behavior of the developed FFL and conventional FMLs.

## 2. Materials and Methods

**Figure 1:**
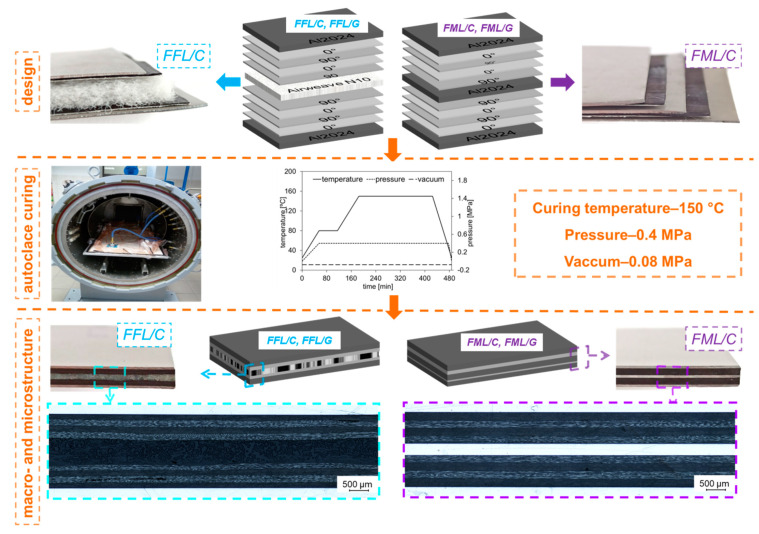
Lay–up scheme of FFLs and FMLs.

Two different types of laminates were examined to determine the impact resistance: Fibre foam metal laminates (FFLs) and conventional fibre metal laminates (FMLs). The laminates used in this study were manufactured of 2024 T3 aluminium alloy sheets (0.3 mm of thickness), and high strength carbon epoxy unidirectional prepregs (0.2 mm of thickness), and composite prepreg based on S2 high–strength glass fibres (0.2 mm of thickness, NTPT, Renens, Switzerland). The lay–up scheme of both types of laminates was 3/2 (three metal layers alternated with two composite layers, see Figure 1), while in the fibre foam metal laminates, the middle aluminium layer was changed to Airweave^®^ N10, a heavyweight non–woven polyester foam structure (AirTech International, Huntington Beach, CA, USA). In order to ensure a better metal–composite adhesion, the surfaces of aluminium sheets were anodized in chromic acid (CAA) and coated with an EC 3924B corrosion-inhibiting structural adhesive primer (3 M, St. Paul, MN, USA).

Laminates were manufactured using an autoclave (Scholz Maschinenbau, Coesfeld, Germany) at the Department of Materials Engineering at the Lublin University of Technology. The cure cycle was carried out at a heating rate of 1.2 °C/min up to 80 °C and held at this temperature for 1 h (pre–curing step), then the temperature was increased to the nominal curing temperature of 150 °C at which the laminates were held for 4 h. Finally, they were cooled down to room temperature at a rate of 2 °C/min (see Figure 1). The pressure and the vacuum used were 0.4 and 0.08 MPa, respectively. Due to the fact that in the autoclave process the prepreg excess resin is removed beyond the prepreg and the sheets block the flow (where is only an edge effect), in the fibre foam metal laminates, the foam component absorbs the excess of matrix from the prepreg, thus it was infiltrated and integrated with the laminate. During the study, aluminium-carbon foam and aluminium–glass foam laminates will be referred as FFL/C and FFL/G, respectively, while aluminium–carbon laminates and aluminium–glass laminates are referred to as FML/C and FML/G (see Table 1).

Table 1 shows the stacking configurations, thickness (*T*) and areal density ($\rho_{A}$) of FMLs and FFLs investigated in this study.

The comparison of microstructures (see Figure 1) shows structural differences in the laminates. In the case of FML/C and FML/G, there is a clear boundary between the carbon or glass composite and the aluminum layer. Instead in the case of FFL/C and FFL/G laminates the internal foam melded with the composite layers.

### Impact Test and Damage Assessment

Specimens were subjected to low–velocity impacts following the guidance of the ASTM D7136 standard using a drop–weight impact tester (Instron Dynatup 9340, Instron, Norwood, MA, USA). A hemispherical impactor tip with a diameter of 12.7 mm (0.5”) was used with a mass of 2 kg (5–10 J) and 4 kg (15–20 J). The samples dimensions were 100 × 150 mm. The hybrid laminates were tested at 5, 7.5, 10, 15 and 20 J of impact energy. The energy range was chosen to achieve a variety of failures from small matrix cracks to catastrophic failure of the laminate. Parameters such as force, maximum force, damage area, displacements and bending stiffness were determined. In order to compare experimental values of impact process between tested materials, the specific force (*F*/$\rho_{A}$) coefficient was used. In physical terms, areal density ($\rho_{A}$) is the mass per unit area. In the case of tested laminates, the ratio considers the density of laminate and their thicknesses (see Equation (1)):

(1) $$\frac{F}{\rho_{A}}=\frac{F}{T}\times \frac{1}{\rho }\;\left[N/\frac{kg}{m^{2}}\right]$$

where: *F*—force [N], $\rho_{A}$—laminate areal density [kg/m^2^], *T*—laminate thickness [m], ρ—laminate density [kg/m^3^]

After the impact, the specimens were subjected to a non–destructive testing using through transmission phased array (*TTPA*), in order to measure the damage and its shape. The *TTPA* analyses were made using an OmniScan MXII ultrasonic defectoscope and TomoView Inspection software (Olympus, Tokyo, Japan). Immersion ultrasonic testing was performed with the use of piezoelectric heads having a frequency of 5 MHz. More details of the technique used can be found in the [39,40]. The damage area was determined using results from *NDT* testing and using Image-Pro Plus 6 software. The *NDT* methods for damage analysis of various materials are used often, as well as validation by using microscopical observations. This technique was used to identify the dominant modes and nature of the damage in the laminate non–impacted side, as well as in the cross–sectionsof the laminated. A detailed analysis of the damages was conducted using a scanning electron microscope (NovaNanoSem, FEI, Hillsboro, OR, USA). It was carried out using secondary electron (*SE*) imaging under 3.0 and 30.00 kV accelerating voltage conditions and a low vacuum (100 Pa) was employed. The samples were previously coated by sputtering using a Quorum Technologies sputter coater model Q150T ES (QUORUM, Lewes, UK).

## 3. Results and Discussion

### 3.1. Force–Time Curves

**Figure 2:**
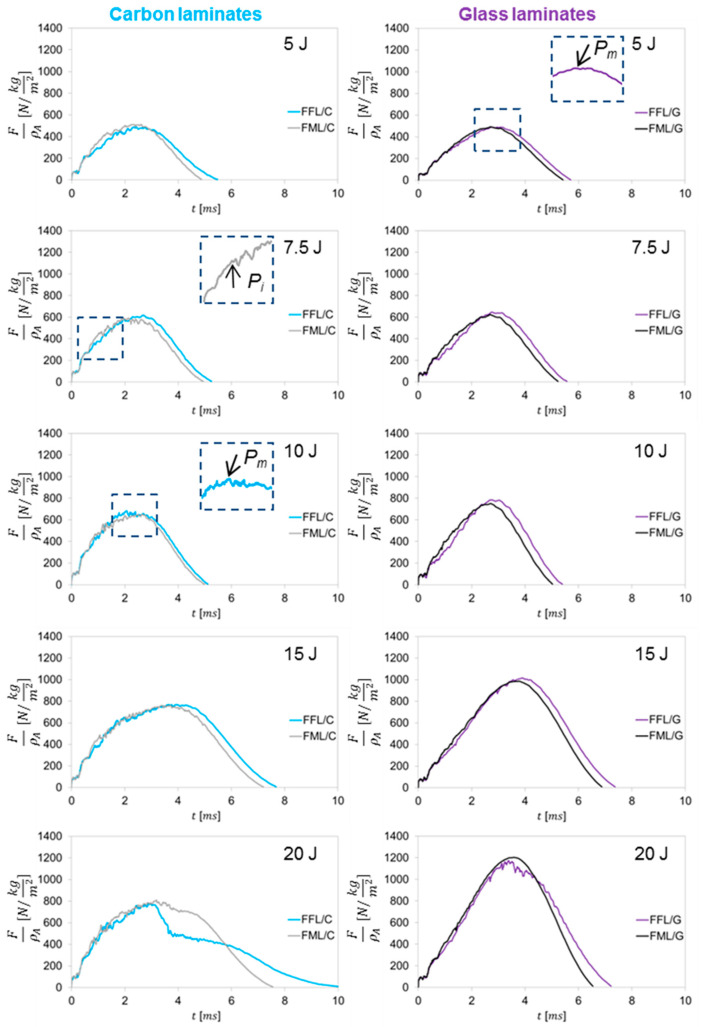
Force–time curves of FFLs and FMLs laminates after low–velocity impact in range of 5–20 J.

The impact response of fibre foam metal laminates and fibre metal laminates at impact energies ranging 5 J–20 J is presented as force–time (*f*–*t*) histories (Figure 2). Carbon and glass fibre metal laminates present an increasing trend of the force as the impact develops, the trend continues up to a maximum value, where the force start to decrease. The maximum force (*P_m_*), corresponds usually with the maximum displacement, in the cases without laminate perforation. Hence, the maximum force at the maximum displacement corresponds with the maximum energy absorbed, beyond this point the laminate rebounds and turn over the elastic energy to the impactor. Usually, reaching the *P_m_* point indicates the initiation of damage and its subsequent propagation which reduces the stiffness [9,34,41]. The force–time plots are characterized by a sudden drop in force overtime before reaching the *P_m_* point, which is especially visible in aluminum–carbon laminates.

In FFL/G and FML/G laminates at impact energies ranging from 5 J to 20 J, but also in FFL/C and FML/C from 5 J to 15 J the curves have a symmetrical shape, corresponding to a non–perforated behaviour of the specimens. With regard to aluminium–carbon foam laminates, the maximum force is up to 4% higher than for FML/C laminates. Similar observations can be noted in the case of glass laminates, where a comparison of maximum force values shows that there is a noticeable increase in the maximum force of aluminium–glass foam laminates. This is a sign of force–carrying ability related to the laminates stiffness reduction in fibre metal laminates, which is connected with a fibre crack initiation of the bottom layer [9]. In some cases, *P_m_* is even higher by 5% for aluminium–glass foam laminates, which gives an average increase in maximum force by 2.6% in comparison to aluminium–glass laminates. The fibre foam laminates have a similar or even higher impact resistance with a lower areal density. The impact at 20 J for FFL/C and FML/C, shows a sudden drop in force after reaching the maximum force, and it suggests a perforation of both types of carbon–based laminates. In the case of glass–based laminates, the crucial changes are visible at the impact energy 20 J, where the curves of FML/G laminate have a symmetric shape without any fluctuations, but in the case of FFL/G laminate, a significant drop of force was observed. In this situation, material loses resistance to the penetrating intender. 

In terms of contact time between the specimen and the impactor tip, some differences were observed. It was noticed that fibre foam laminates interact with the intender for a longer time than fibre metal laminates. This difference was on average 12.3% for FFL/C laminates and 7.3% for FFL/G in comparison to FML/C and FML/G. The FFLs with a lower areal density and hence lower inertia produce a more efficient distribution of the stress during the impact. On the other hand, the FMLs, with a lower contact time, may be characterized by a better ability to return the laminate to the elastic range after reaching its maximum force and deflection. Similar observations were confirmed by other authors [13,41,42].

**Figure 3:**
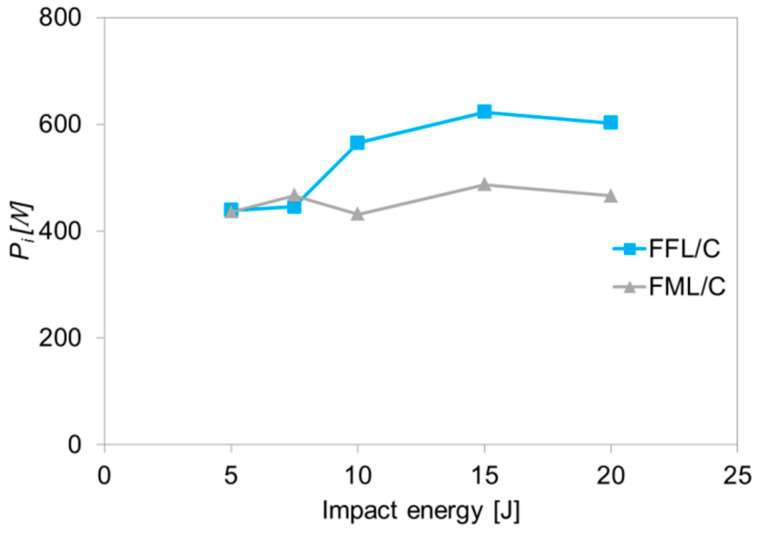
*P*_i_ of laminates depending on impact energy.

Based on the analysis of *f*–*t* curves of FFL and FML laminates, it was exhibited that glass laminates do not show sudden and temporary drops in force. This is due to the maintaining of the local structure integrity and indicates the laminate’s ability to carry a further impact force. In the case of carbon–based laminates, both FFL/C and FML/C were characterized by a local decrease in force during the growth stage before reaching the maximum force (Figure 2). This is characteristic for this group of materials due to the high brittleness of carbon fibres [4,6,12]. Figure 3 presents the force of damage initiation (*P*_i_) for each of the impacts as a function of the impact energy.

It can be observed that up to 7.5 J *P*_i_ is similar for both types of laminates. Beyond this energy, the FML/C presents a lower value than the FFL/C. This phenomenon could be related to the degradation of the foam layer. Due to the fact that *P*_i_ is considered to be an initiation of layer cracks, it should be expected that in the low energy range the foam layer in FFL/C will degrade earlier than the carbon layers in FML/C laminates. At higher impact energies (above 10 J) it was observed that *P*_i_ is around 30% higher for FFL/C laminates than for FML/C laminates. Thus, it is clear that *P*_i_ is a parameter of each plate type related with the initiation of damage in the specimen (crack initiation [12]). Based on the initiation force parameter, it can be stated that aluminium–carbon foam laminates have a higher impact resistance than aluminium–carbon laminates under low–velocity impact. The above can suggest that the foam component in the FML construction increases the ability against the perforation starting point, especially in the case of carbon–based laminates which are sensitive to brittle cracking. This phenomenon will be assessed in the further evaluation of the damages of both types of laminates

### 3.2. Force–Displacement

**Figure 4:**
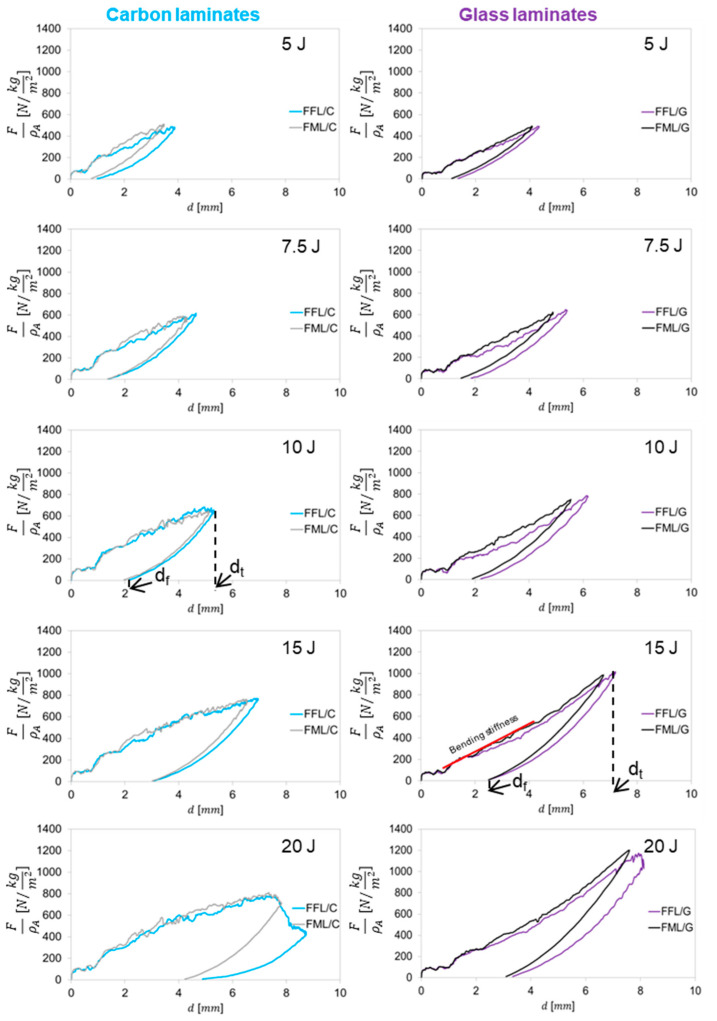
Force–displacement curves of glass and carbon laminates with and without internal foam component.

Figure 4 shows the specific force–displacement (*f*–*d*) curves for the all the laminates at impact energies ranging from 5 J to 20 J. Based on the analysis of the force–displacement curves: total deflection (*d_t_*), final deflection (*d_f_*) and bending stiffness (*K*) can be determined. However, only based on the shapes of *f*–*d* curves it was noted that some differences between fibre foam metal laminates and conventional fibre metal laminates occurred independently of impact energy.

**Figure 5:**
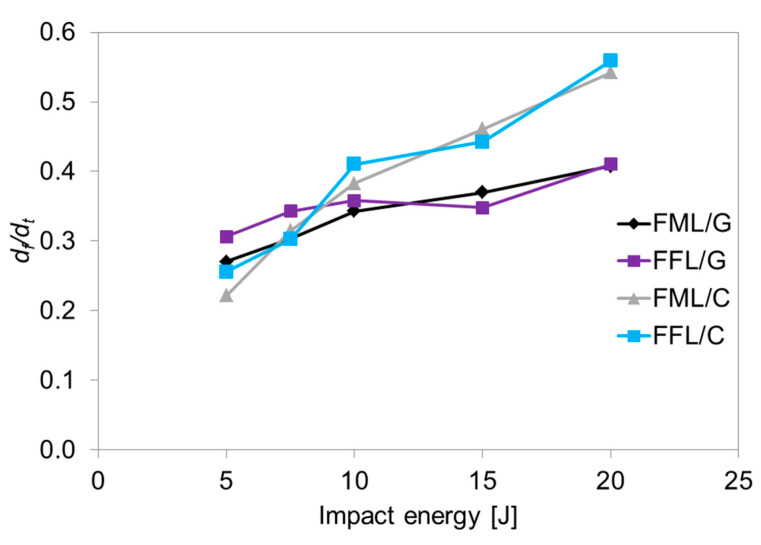
The relation between *d_f_/d_t_* coefficient and impact energy.

A comparison of the *f*–*d* curves of glass–based laminates shows slight changes in force over time, both during the increase and decrease stages. In tested laminates, a higher slope is visible for fibre metal laminates than for fibre foam metal laminates. The total displacement, as well as the final displacement of the laminates caused by the impactor in all the impact energies, are higher for FFL than for FML. The difference between final deflection and total deflection of FFL/C averages 12% and 8% compared to FML/C laminates, while for FFL/G this difference is 14% and 8.5% in comparison to FML/G laminates. This difference is due to the energy absorption and dissipation mechanism between laminates. It was noted that the final deflection (*d_f_*)is independent of the presence of foam in the structure of the laminate, especially in the case of carbon-based laminates (Figure 4). The analysis of the coefficient *d_f_*/*d_t_*, which describes the laminate capacity to accumulate energy through the elastic deformation with correlation to permanent deformation [43] was presented in Figure 5, it can be observed a positive trend with the impact energy.

In most cases, a higher value of the *d_f_*/*d_t_* ratio was noted for FFL/C, on average by 3.5% in comparison to FML/C. The lowest *d_f_/d_t_* coefficient for FFL/C was 0.26, while for FML/C it was 0.22 at the impact energy 5 J, while at the energy 20 J, this value increased to 0.56 and 0.54, respectively. The contribution of permanent deformation in the total deformation increases from 20% to over 50% in the energy range from 5 to 20 J, both for FML/C and FFL/C laminates. This is due to the failure progression with increasing impact energy. At low energies, the energy is accumulated by a deformation in the elastic range. When the impact energy reaches the maximum elastic energy absorption the energy absorption process by permanent deformation starts (plastic deformation of metal layers and failure of composite and foam layers). Similar observations were noted in the case of glass laminates, where aluminium–glass foam laminates were characterized by higher *d_f_/d_t_* values. The difference between final deflection and total deflection of FFL/G averages 14% and 8.5% compared to FML/G laminates. The *d_f_/d_t_* coefficient in FFL/G laminates has a higher value at all energies except 15 J and is higher by an average of 5%. The lowest value was 0.27 for FML/G and 0.31 for FFL/G, while the highest value equaled 0.41 and it was the same in both cases. Simultaneously, the FFL/G with an impact energy from 10 J to 15 J has a different trend in Figure 5. This phenomenon can be related to the changing of the damage mechanisms which will be deeper analyzed in section of damage analysis.

**Figure 6:**
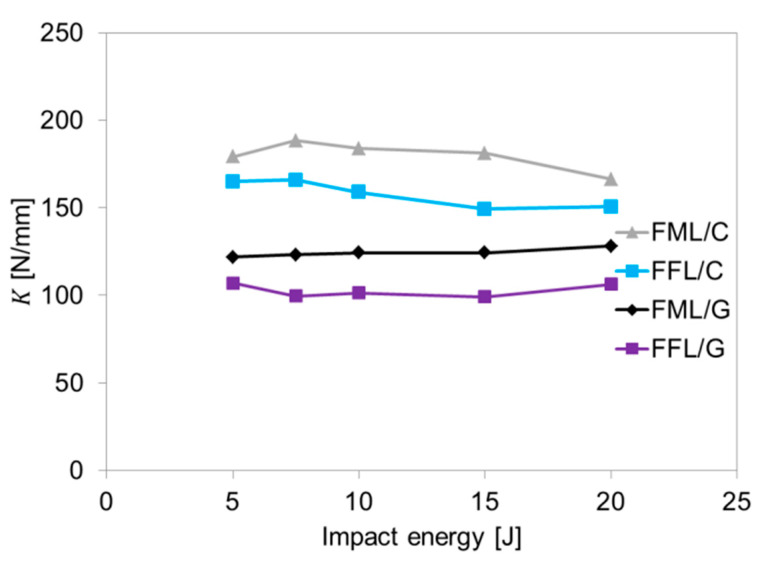
Bending stiffness of investigated laminates under various impact energies.

The difference between the values of the *d_f_*/*d_t_* coefficient of carbon- and glass-based specimens is due to the deformability of the fibres; carbon fibres have an elongation from 1.5 to 2% and glass fibre around 5.5% [44]. The noted differences in *d_f_*/*d_t_* coefficient between conventional fibre metal laminates and fibre foam metal laminates may indicate the influence of the foam layer on the deformation process, energy accumulation ability and plate elasticity (see Figure 5). Therefore, the bending stiffness (*K*) of laminates was complementarily analyzed in relation to areal density (Figure 6). The bending stiffness parameter describes the impact resistance and delamination vulnerability [42,43,45].

The analysis of the results shows that using foam layer instead of metal layer causes a significant decrease in bending stiffness of the laminates. The *K* in general is independent on the impact energy, but it is a feature of a laminate (when keeping comparable: thickness, geometry and boundary conditions of the bending test). However, the *K* parameter can be used for comparison between conventional fibre metal laminates and laminates with foam. The average value of the bending stiffness for FFL/C laminates is 157.9 N/mm (standard deviation, SD 6.9 N/mm) and it is 13.9% lower in relation to FML/C laminates (179.7 N/mm, SD 8.3 N/mm). In the case of FML/G, K is on average 124.3 N/mm (SD 2.4 N/mm), which is over 17.4% higher than FFL/G (102.7 N/mm, SD 3.7 N/mm). Moreover, this difference may be a result of the lower proportion of metal layers in fibre foam metal laminates [42]. Literature data show that foam sandwich structures exhibit higher bending stiffness, but that is in the case of conventional, thicker and rigid foams like PVC [23]. The used polyethylene foam is elastic, which was important for the simultaneous compression and infiltration by a composite matrix. Nonetheless, it should be noted that the bending stiffness differences between FML and FFL correspond with results from force–displacement curves where it was observed that fibre foam metal laminates were characterized by greater deflection than fibre metal laminates (see Figure 4) reaching higher energies and hence more energy. Consequently, a greater deflection of the laminate is possible, which causes a greater stress concentration in the impact region, and higher delamination due to shear between the layers [16].

### 3.3. Energy Absorption

**Figure 7:**
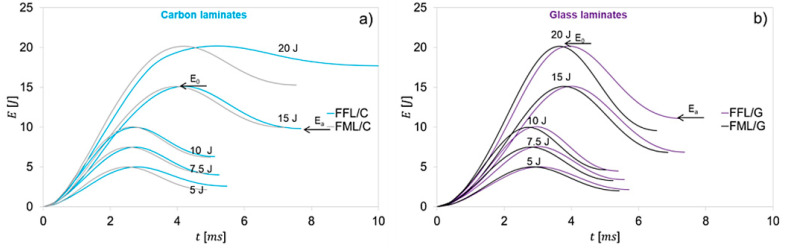
Experimental Energy–time curves of (**a**) FFL/C, FML/C and (**b**) FFL/G, FML/G impacted with the energy ranging from 5 J to 20 J.

The energy–time curves (*E*–*t*) of impacted laminates are presented in Figure 7. First of all, it can be stated that the energy as a function of time increases until the maximum energy (*E_0_*) is reached, which corresponds to the kinetic energy of the impactor. Then, the energy decreases to reach the energy absorbed (*E_a_*) by the laminate.

The analysis of the *E*–*t* curves show higher energy absorption *E_a_* by the fibre foam metal laminates in comparison to the fibre metal laminates. For FFL/C the value of absorbed energy is on average 6.3% higher, while for FFL/G laminates this value is on average 4.9% higher, in comparison to carbon and glass fibre metal laminates, respectively.

The difference in the amount of energy absorbed by laminates is a result of the initiation process and propagation of the damage in the foam instead of metal or composite layers. Foam core is damaged by crushing and shearing, which results in delamination at the foam–composite interface and foam failure [26,27,30,32]. Furthermore, based on the absorbed energy and impact energy, the coefficient of restitution (COR) was determined, which enables the assessment of material failure mode at different impact energies [46,47]:

(2) $$\mathrm{COR}=\sqrt{\frac{E_{a}-E_{0}}{E_{a}}}$$

where: $E_{a}$—impact energy, $E_{0}$—energy absorbed by laminates 

**Figure 8:**
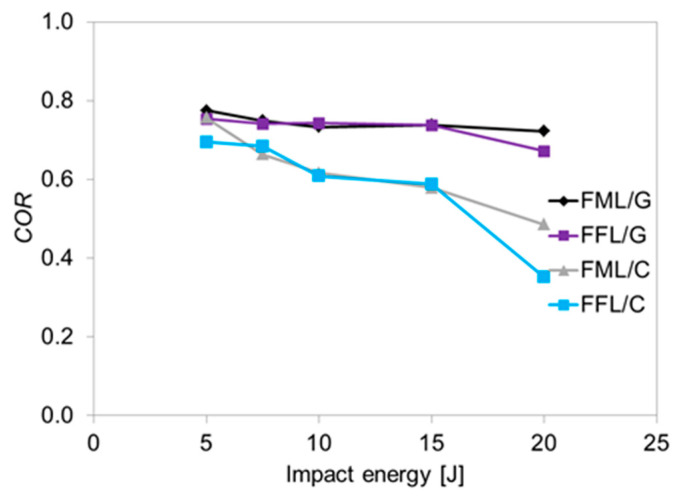
COR coefficient vs. impact energy for all laminates.

The results obtained based on the COR coefficient depending on the impact energy are shown in Figure 8.

The analysis of COR coefficient shows that it decreases with increasing impact energy for all investigated laminates. When the coefficient is close to 1 the specimens is intact or the main damage is delamination, as the impact energy increase the driven damage changes to fibre and metal damage, $E_{0}$ increases, and hence the COR decreases. Finally in the perforation all the kinetic energy of the impactor is absorbed or dissipated and COR is zero [47]. The glass-based laminates are characterized by the highest values of COR, although FFL/G has comparable values in the range of 5–15 J, which suggests a similar nature of the damage. Despite the use of the foam layer instead of an internal metal layer, the damage stage sequence is similar in both types of laminates. In the investigated energy range, glass fibres have a higher contribution of energy absorption due to their high elongation capacity (5.5% strain to failure). This delays the initiation of the fibre cracking process and increase the capacity of energy absorption by the remaining laminate components. In the case of carbon–based laminates it was noted that FFL/C are characterized by a similar value of COR coefficient in comparison to FML/C laminates so there were no significant changes in the failure nature. A relevant difference was observed at the 20 J, because the value of FFL/C was 27.5% lower than in FML/C what suggests extensive internal damage. 

### 3.4. Damage Analysis

**Figure 9:**
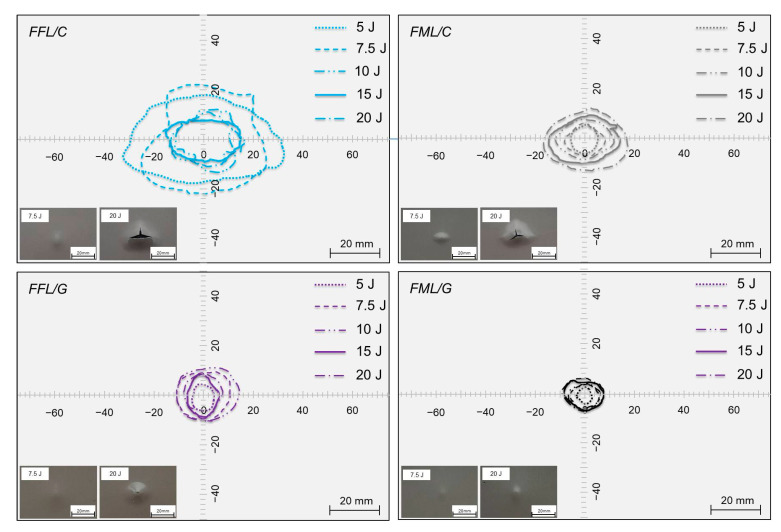
Damage area and non-impacted side of FFLs and FMLs.

In the Figure 9 the macroscopic view of the non-impacted side of investigated laminates and internal damage area (*D_a_*) were presented.

The analysis of the non-impacted side of the tested materials indicates that in the range from 5 J to 20 J some changes to the structure occur. Depending on the type of material and impact energy, various impact behaviors of the laminates were observed. 

**Figure 10:**
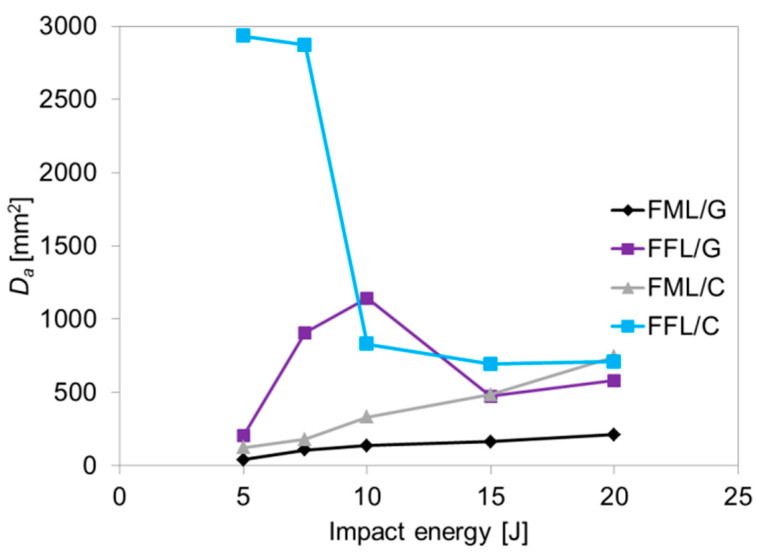
The comparison of damage area of FFL/C, FML/C, FFL/G and FML/G.

The analysis showed a progressive degradation of the laminates and the appearance of subsequent forms of damages as the impact energy increase: from local deformation, metal cracks initiation, to laminates perforation. The first crack at the bottom side in both types of carbon–based laminates was observed at 7.5 J. In FFL/C the crack is smaller than in the FML/C, 2 mm vs around 5 mm long, the foam layer absorbs energy and prevents the cracking of the bottom metal layer. The second crack appears in impacts at 15 J, denoting the beginning of the perforation, whereas the partial perforation (cracks of bottom and top metal layers) occurs at the energy of 20 J. The analysis of the non-impacted side of glass-based specimens laminates does not show visible external damage in the range between 5 and 15 J of impact energies. The above–mentioned energy range is only characterized by a local deformation, which is more pronounced and round in the case of FFL/G laminates. The initiation process of the first crack of the bottom side of glass laminates was observed at the impact energy equal 20 J for FFL/G laminates. However, the internal structure may have been damaged as a result of delamination, fibre cracks, or matrix cracks. There were no significant differences in the shape and location of the damage between the laminates. The shape of delamination in all tested laminates is nearly round, which is characteristic for FMLs due to the isotropy of metal layers, the same as foam layer in modified laminates. Only some elliptical shape can be seen in the case of carbon–based laminates due to the higher stiffness of the carbon fibre and its damage due to its low fracture strain [12,48,49]. Simultaneously, FFL/C damage area shape can be characterized by a less regular shape (see Figure 9). In some cases (e.g., 7.5 J and 10 J) the damage area has an undefined shape. Some bends in the curvature of the delamination boundary are noticeable. These differences may be as a result of isotropic metal layers, which do not have a preferred damage propagation direction. Moreover, some differences in the magnitude of damage area between FFLs and FMLs were noted. The most significant internal damage area can be observed for FFL/C laminates. The increase, in the damage area of foam laminates in comparison to conventional fibre metal laminates suggests an increase in the effect of energy dissipation in foam component, and its contribution to the total energy absorption by its damage [19,26,30]. In the Figure 10 shows the comparison of the damage area values of tested laminates.

It was noticed that the damage area of FFL/C laminates decreases with increasing impact energy, while in the case of FFL/G the relation changes from an initial increase (up to the energy of 10 J) to a further decrease (see Figure 10) (extensive testing campaign should be done to focus on this trends). The above trend is different in fibre metal laminates, where the damage area increases with the impact energy until a quasi–constant value is attained [3]. The high value of damage area in the case of FFL/C, in particular under impact energys of 5 J and 7.5 J, is due to a change in the energy absorption mechanism. At low impact energies, the dominant failure mechanism is the formation of delaminations and matrix cracks and not fibrs cracking due to high local stress concentration (as in the case of FMLs). Higher impact energies produce higher deformation and the initiation of catastrophic failures (fibre cracking, metal cracking) of both types of laminates. In the case of FFL/G a significant growth of failure was observed for impact energy range 5 J–10 J, where the increase in the damage area was 8 times more extensive than in the conventional aluminium–glass laminates. The reason for the above fact is also the influence of foam component, and its damage. However, after 10 J of impact energy, a successive decrease in the damage size was observed, which is a result of the increasing importance of other laminate components (metal, glass fibres) in the process of energy absorption and stress distribution. 

**Figure 11:**
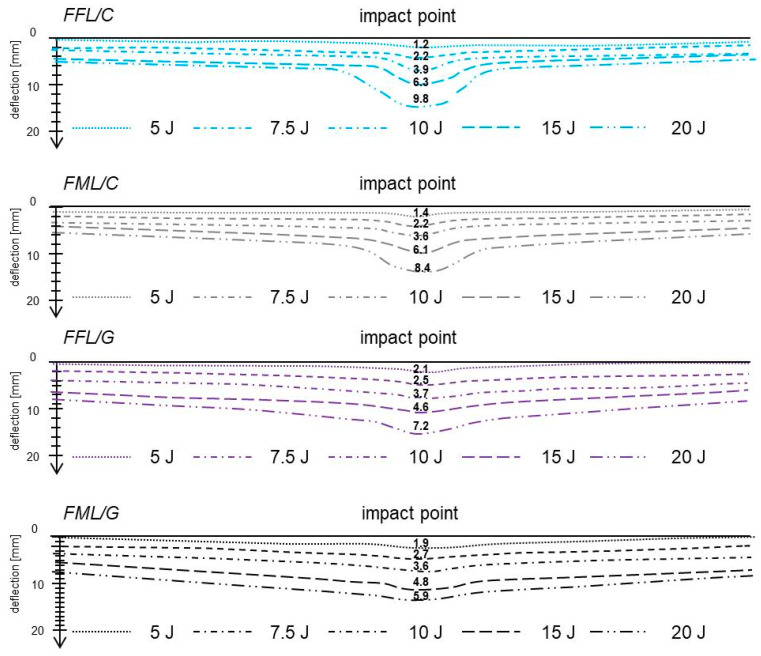
Deflection profiles of the top edge of laminates after impact.

Due to the importance of deflection in damage initiation and observed energy absorption mechanisms in fibre foam metal laminates and conventional fibre metal laminates, the deflection profile (in cross–section through the impact point) of laminates are presented in Figure 11.

It was observed that in the carbon–based and glass–based laminates deflection increases with increasing impact energy. In general, higher values of deflection were measured in carbon laminates than in glass laminates, due to the low strain to failure of carbon fibres (cracked fibres reduce significantly the laminate stiffness, which causes a greater energy absorption of the metal components, i.e., causes greater plastic deformation of the metal layers) [3]. The values of permanent deformation of foam fibre metal laminates and conventional laminates are not significantly different. The permanent deflection varies between the 1.2 and 9.8 mm for FFL/C, and between 1.4 and 8.4 mm for FML/C. In the case of glass–based laminates, the values vary between 2.1 to 7.2 and 1.9 to 5.9 for FFL/G and FML/G respectively. The most significant difference occurs in the shape of the deflection profile. In fibre metal laminates deflection at the impact point has a characteristic elongated shape, while in the fibre foam metal laminates the deflection at the impact point is more flattened and not so pronounced, however, it is more deflected along their entire length. This is due to the linear elastic characteristics of the foam, it does not deform locally but bends in a larger area promoting a more global deflection. Which is consistent with the larger damage area in foam laminates and plastic deformation of the metal. As a consequence, the accumulation of the energy is higher in FFLs, but when the elastic deformation of foam reach is limit, brittle cracking of this layer occurs in a larger area (mechanisms of energy absorption). In the case of the metal layer, plastic deformation is one of the energy absorption mechanisms, and delamination occurs.

### 3.5. Failure Analysis

Besides the permanent deflection profile of laminates, the cross–sections through the impact point were analyzed in terms of failure mechanisms. The comparison of the failure mechanisms in carbon–based and glass–based laminates was shown in Table 2 and Table 3. The tables present the example of the damage inside the specific laminate after impact (failure type). The point marked in the specific position put in the cross of the laminate type and failure type means that the damage was observed. The differences between FML and FFL were additionally marked with the color box.

The analysis of the cross–sections in the impact area indicates some differences between conventional fibre metal laminates and fibre foam metal laminates at impact energies between 5 J and 20 J. Based on the failure analysis a variety of damage types were observed depending on the value of impact energy and laminate type, including delaminations, fibre and matrix cracks, or metal cracks. The delaminations are the main failure mode in fibre metal laminates under low–velocity impact, which is the result of interlaminar shear at the interfaces of the layers [9]. Moreover, literature data confirms that delaminations, at the interface between composite and foam, are also an important type of damage for sandwich structures [26,27,33]. The analysis reveals that delaminations in all interfaces (foam–composite, composite–composite (0/90) and metal–composite) could be observed in FFL/C and FML/C laminates for all of the impact energies. The difference appears in the fibre cracks at low impact energy (5 J), in this case the FML/C does not show any crack, may be due to the deformation of the foam. More extensive delamination in FFL/C is liable for the energy absorption, which in FML laminates is absorbed by fibre breakage as a result of intense, but local deformation (in the area of contact with the indenter). Foam cracks are a characteristic failure for materials including foam, which was confirmed in numerous studies [27,49,50]. Foam laminates can absorb a greater amount of energy during deformation by foam cracks and delaminations at the foam–composite interface. All these observations are in accordance with the trend of damage area in Figure 10. 

The damages of tested glass laminates change significantly as a function of the impact energy. The analysis of cross–sections of glass laminates shows similar types of damage like in carbon laminates except for catastrophic damage–perforation. 

Based on the detailed analysis of cross–section in the impact area of fibre foam metal laminates based on the glass fibres, it was noted that delaminations at the foam composite, metal–composite and composite–composite interface are the only type of damage at the energy 5 and 7.5 J, fibre cracks appear additionally above energy 7.5 J. Similar behaviour of the aforementioned in carbon based specimens could be concluded related with the delamination damage, the main difference in this case is the observation of fibre cracks at the energy from 5 to 20 J in FML/G laminates, corresponding with the literature data [3,51]. The reason for the difference in the types of damage in the low energy between FFL/G and FML/G is also the influence of foam component due to its energy absorption process. It suggests that mainly the foam layer was involved in the process of absorbing energy, through crushing and cracking but also through delamination at the foam–composite interface [30]. The first crack of the bottom metal layer appears at 20 J of impact energy for FFL/G laminates, whereas through the investigated energies no metal crack in the FML/G laminates was observed. The metal crack in FFL/G laminates may be a result of internal delaminations, and fibre or matrix cracks.

### 3.6. Fracture Analysis

**Figure 12:**
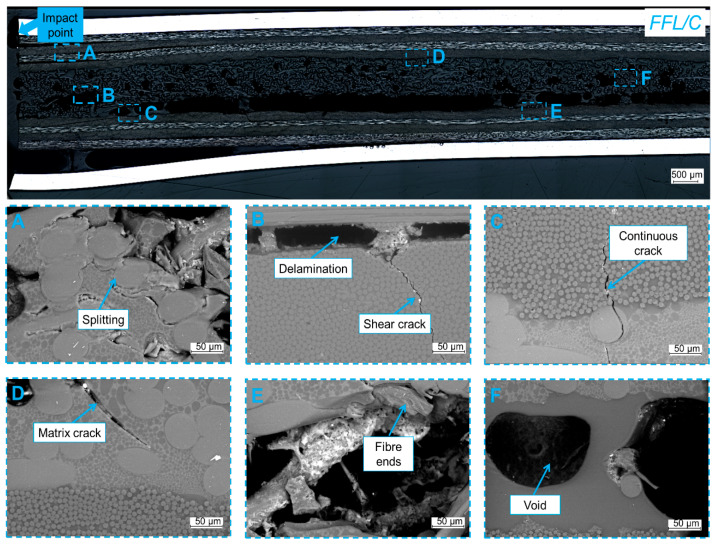
Micro–failure damage typesof FFL/C after impact ((**A**) splittings, (**B**) delamination and shear cracks, (**C**) continuous intralaminar crack, (**D**) matrix cracks, (**E**) fibre breakage, (**F**) void).

In order to determine the micro–failure mechanisms characteristic of fibre foam metal laminates, fractography was performed. Besides general delaminations between composite layers in laminate and cracks of specific elements of laminate, the micro–failure analysis shows that those types of failure occur in a mixed manner [3,4,9,13]. In addition to matrix cracks and interface cracks, also can be observed matrix shear cracks, splitting on the matrix–fibre interface, fibres pull out, and others [6,8,34,52,53]. Replacing the inner metal layer with foam with lower stiffness and drastically different properties, may lead to the creation of new and subsequent damage mechanisms responsible for energy absorption. They mainly include the interaction at the composite–foam interface. This is due to the fact that the failure between metal and composite or composite and composite remains unchanged, as it was shown in the damage analysis on cross–sections. The detailed analysis of micro–failure of the foam component of FFL/C was presented in Figure 12.

**Figure 13:**
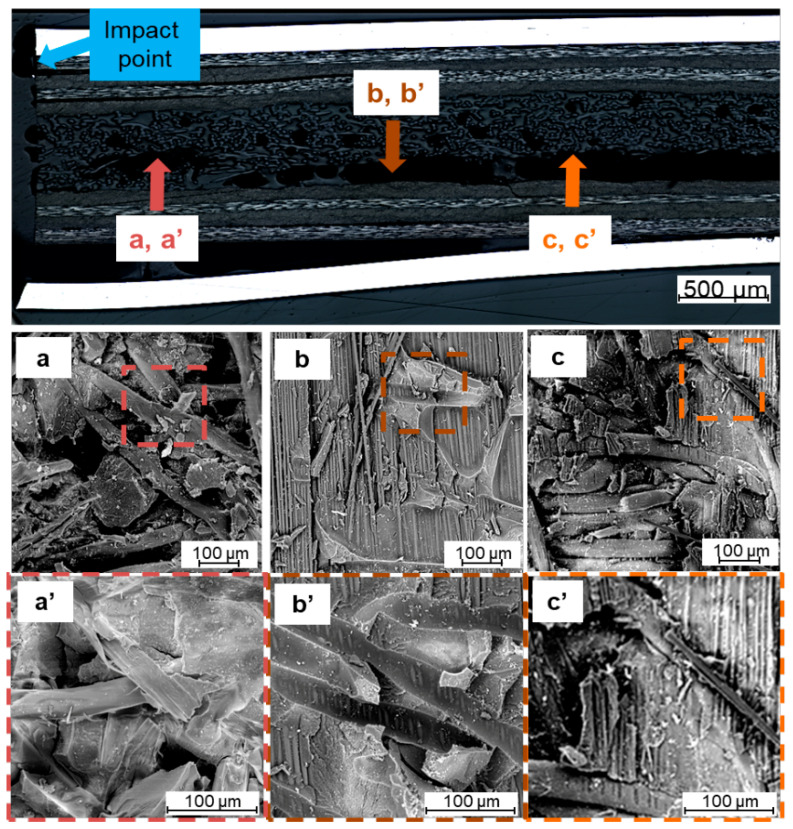
Morphology of the failure of foam (**a**,**a’**) and foam–composite interfaces (composite surface (**b**,**b’**) and foam surface (**c**,**c’**) in Fibre Foam Metal Laminates after impact.

The observations of the failure of FFLs indicate some damages characteristic of classic fibre metal laminates like fibre–matrix splitting (Figure 12A), shear cracks (Figure 12A,C), but also matrix cracks (Figure 12D) and delaminations (Figure 12B). It was observed that interlaminar delaminations were found between layers oriented (0) and (90), which is common with conventional FMLs [1,9]. It is worth noting that there were no delaminations at the top composite and foam interface, only at the foam and bottom composite interface. Moreover, the delamination is not present through the entire length, there are some places where no delamination was observed, and where the foam structure is connected with the lower composite layer. Furthermore, some delaminations or cracks inside the foam component were observed, there are visible fibre ends (Figure 12E) and epoxy matrix cracks (Figure 12D). The microstructure analysis revealed that a path of matrix cracks is connected with matrix–fibre separation [51] while it was observed that polyester fibres have the feature of blocking the development of cracks (Figure 12C). Continuous crack through composite–foam layers was noted in Figure 12C, it seems that a common matrix in the foam and composite or the lack of young modulus mismatch does not result in shear at the boundary of the composite foam layers. Besides the translaminar and intralaminar matrix cracks, the delamination at the various interfaces and inside the foam was found. Some empty spaces observed between the foam structure are known from the literature data as voids and they do not transfer stresses (Figure 12F). The detailed analysis of mechanisms of interaction of foam component and composite adjacent layers during failure propagation is presented in Figure 13. The authors focused on the morphology of the structure at the foam–foam, foam–composite, and composite–foam interface.

In the case of damage inside the foam, it was shown that the polyester fibres are infiltrated with matrix from composite layers. The path of cracking in polyester fibres is undefined. They cracked at different heights, which can indicate there was a multidimensional cracking (Figure 13a,a’). The fibre cracks occur without prediction, and at consistently scattered places. The degradation of the volume of foam in FFL means, that besides the deformation type of energy absorption of foam the damage formation and propagation also is responsible for partial energy absorption, which corresponds with the higher energy absorption capacity of FFL in comparison with conventional FML, especially at low impact energy range. The detailed analysis of the delaminated composite–foam interface (composite surface) shows foam matrix residue on the composite layer. On the composite delaminated surface (Figure 13b,b’), imprints of polyester fibres can be observed. Such phenomena show the good coherence in the epoxy impregnated in the foam and the remain in the composite. In practice, crack paths are not located ideal at the interface straight line but are going through the matrix volume from composite fibre to polyethylene fibre (crack migration favors energy absorption [52]). Analysis of the delaminated composite–foam interface (foam surface) (Figure 13c,c’) shows smooth fibre imprints with the delamination of carbon fibres from the matrix. This suggests that the carbon fibres were entirely separated from the matrix [52]. Furthermore, single carbon fibres were observed on the foam surface, which indicates splitting of the composite layers and fibre cracks, producing fibre bridging and hence energy absorption. However, there are few rests of fibres, which suggests that regardless of the structure examined (conventional FRP, conventional FML, or FFL laminates), the matrix remains the least fracture-resistant component of composites under impact conditions [12,52].

## 4. Conclusions

Based on the state of art related to impact features of fibre metal laminates and sandwich structures, it was assessed that it is possible to improve the impact resistance of hybrid structures by mixing these two types of materials. Therefore, a hybrid FML structure, containing, besides composite and metal layers, a polyester foam layer infiltrated with resin obtained from prepreg composite during curing (fibre foam metal laminates) was prepared. The goal was to create a material that will combine the superior properties of FMLs and foam sandwich structures in terms of the impact resistance and simultaneously will have a lower density (minimum 5%) and fewer disadvantages related to the manufacturing

The impact performance of a modified fibre metal laminate was studied. For this purpose carbon and glass fibre metal laminates have been manufactured switching its metal core by a foam one. Its performance under impact was compared with its homologous conventional fibre metal laminates. The main conclusion of the research could be summarized as follows:

(1) The inclusion of the foam core leads to similar or higher impact resistance and significant changes in impact behavior in the studied energy range. The inclusion of a foam core does not reduce significantly the impact perforation resistance of the specimens.
(2) The amount of absorbed energy by the foam fibre metal laminates was higher than the conventional ones. The after-impact damage analysis and fractography concludes that the energy absorbed by the foam and its influence in the damage apparition in the impact improve the specimen behaviour under impact.
(3) The permanent deflection of the specimens after impact with foam cores is similar to that of conventional specimens. The buckling thread under compression after impact does not increase.
(4) The better adhesion between the foam core and the composite than between the metal and composite plies results in a higher performance in terms of energy absorption of the specimens.

Finally, it can be summarized that benefits can be achieved by using this new type of hybrid. In terms of practical implication, the design, and manufacturing possibilities, as well as properties (density, impact resistance), are major contributions of the proposed solution. In terms of scientific knowledge, the impact behavior of the hybrid structures demonstrate changes in the damage mechanisms. The experimental study performed in this research and the comparison of the results with conventional baseline materials are the first steps in further development of multi-material hybrid thin layer structures.

## Figures and Tables

**Table 1:** Stacking configurations.

Material	Configuration	*T* [mm]	$\rho_{A}\;[\mathrm{kg}/m^{2}]$
FFL/C	Al/CFRP(0/90)_2_/Foam/CFRP(90/0)_2_/Al	2.58	4.68
FML/C	Al/CFRP(0/90)_2_/Al/CFRP(90/0)_2_/Al	2.49	5.01
FFL/G	Al/GFRP(0/90)_2_/Foam/GFRP(90/0)_2_/Al	2.35	4.91
FML/G	Al/GFRP((0/90)_2_/Al/GFRP(90/0)_2_/Al	2.38	5.17

**Table 2:** Types of failure in carbon–based laminates.

Sample	Delamination Foam–Composite Interface (A)	Delamination Metal–Composite Interface (B)	Delamination Composite–Composite (C)	Bottom Metal Crack (D)	Fibre Cracks (E)	Perforation (F)
View of failure type	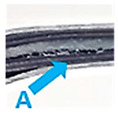	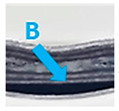	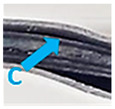	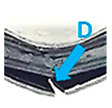	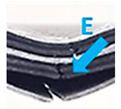	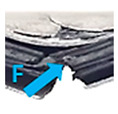
FFL/C 5 J	•	•	•			
FFL/C 7.5 J	•	•	•	•	•	
FFL/C 10 J	•	•	•	•	•	
FFL/C 15 J	•	•	•	•	•	
FFL/C 20 J	•	•	•	•	•	•
FML/C 5 J	not applicable	•	•		•	
FML/C 7.5 J	n.a.	•	•	•	•	
FML/C 10 J	n.a.	•	•	•	•	
FML/C 15 J	n.a.	•	•	•	•	
FML/C 20 J	n.a.	•	•	•	•	•

**Table 3:** Types of failure in glass–based laminates.

Sample	Delamination Foam–Composite Interface (A)	Delamination Metal–Composite Interface (B)	Delamination Composite–Composite (C)	Fibre Cracks (D)	Bottom Metal Crack (E)
View of failure type	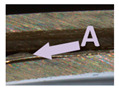	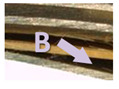	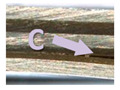	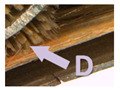	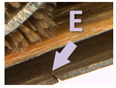
FFL/G 5 J	•	•	•		
FFL/G 7.5 J	•	•	•		
FFL/G 10 J	•	•	•	•	
FFL/G 15 J	•	•	•	•	
FFL/G 20 J	•	•	•	•	•
FML/G 5 J	n.a.	•	•	•	
FML/G 7.5 J	n.a.	•	•	•	
FML/G 10 J	n.a.	•	•	•	
FML/G 15 J	n.a.	•	•	•	
FML/G 20 J	n.a.	•	•	•	

## Data Availability

The data presented in this study are available on request from the corresponding author.
